# Successful long-term management of metastatic clear cell renal cell carcinoma with nivolumab: a case report and literature review

**DOI:** 10.3332/ecancer.2023.1643

**Published:** 2023-11-30

**Authors:** Nicolás Duque Clavijo, Paula A Lara, John Alejandro Murillo Silva, Iván Camilo Triana, Henry Alexander Vargas, Luis Eduardo Pino, Javier Mauricio Segovia, Erick Andrés Cantor

**Affiliations:** 1 Universidad de los Andes, Fundacion Santa Fe de Bogota, Bogota 110111, Colombia; 2 Internal Medicine Department, Santa Fe Fundacion Santa Fe de Bogota, Bogota 110111, Colombia; 3 Internal Medicine Department, Fundacion Santa Fe de Bogota, Bogota 110111, Colombia; 4 Internal Medicine Department, ICCAL, Fundacion Santa Fe de Bogota, Bogota 110111, Colombia; a https://orcid.org/0009-0009-4553-5168; b https://orcid.org/0009-0000-0080-1084; c https://orcid.org/0000-0001-7450-8286; d https://orcid.org/0000-0002-8349-5576; e https://orcid.org/0000-0003-2039-1857; f https://orcid.org/0000-0003-4475-7470; g https://orcid.org/0000-0001-8971-0911; h https://orcid.org/0000-0002-0920-219X

**Keywords:** clear cell renal cell carcinoma, metastatic disease, immunotherapy, immune checkpoint inhibitor, complete remission

## Abstract

In Colombia, renal cancer is a rare condition, with clear cell renal cell carcinoma (ccRCC) being the most prevalent neoplasm. In recent years, immune checkpoint inhibitors (ICI) have been proposed for the management of metastatic disease, as they have shown improved rates of response and long-term survival. Furthermore, they exhibit a favourable tolerance profile, and adverse events causing significant morbidity are infrequent. We report the case of a 61-year-old male patient initially diagnosed with early-stage ccRCC who underwent right nephrectomy in 2009. Six years later, disease recurrence with metastatic compromise was documented, which led to the resection of the L1 vertebral body followed by radiotherapy and maintenance treatment with sunitinib. Due to disease progression, treatment with sunitinib was discontinued. Subsequently, everolimus was initiated as second-line immunotherapy, which was later discontinued due to the appearance of new metastatic lesions. In 2017, the patient was referred to our institution, where a third-line pharmacological treatment with nivolumab was initiated. In 2022, complete remission by positron emission tomography-computed tomography (PET-CT) was evidenced, which has been sustained to date. This case demonstrates the efficacy and safety of ICI in patients with metastatic ccRCC. The case presented is relevant in that it describes the achievement of complete remission in a patient who did not respond to the first two lines of immunotherapy. Given the limited literature regarding the discontinuation of therapy after achieving sustained remission, further research is warranted to explore this topic.

## Introduction

Kidney cancer is a relatively uncommon condition in Colombia, ranking 13th place for incidence among cancers in this country. Its incidence is five times lower than that in the United States and occurs more frequently in men than in women [1, 2]. Referring to primary malignant neoplasms of the kidney, the most common corresponds to Clear Cell Renal Cell Carcinoma (ccRCC), which accounts for approximately 70% of cases of kidney cancer [3]. This histological type is associated with the loss of the short arm of chromosome 3 (3p), which contains the von Hippel-Lindau gene (VHL). In fact, loss of both alleles of the VHL gene, either by deletion or by epigenetic silencing, has been found in more than half of patients with this type of renal cell carcinoma [4]. The inactivation of the VHL gene is recognised for its role in disrupting hypoxia-inducible factor function, resulting in the upregulated expression of several genes, including vascular endothelial growth factor [5]. Regarding its clinical presentation, it is worth noting that many patients are asymptomatic in the early stages of the disease, with many of these diagnoses occurring as incidental finding. In fact, only a small proportion of patients present with the classic triad of the disease, consisting of haematuria, flank pain and palpable flank mass [5]. As for the prognosis of patients with kidney cancer, it is well known that it is highly dependent on the stage of the disease. The overall 5-year survival is estimated to be 76% for all stages. However, while a very unfavourable prognosis is estimated for patients with distant metastases, with only a 14% overall survival (OS) rate at 5 years, those with disease confined to the kidney or with regional dissemination have much higher 5-year survival rates, with rates of 93% and 71% of cases, respectively [6]. Currently, nephrectomy is the mainstay of treatment in cases of localised and regional disease, while immunotherapy and targeted therapy represent the best therapeutic options for metastatic disease when there is curative intent, as chemotherapy and radiotherapy have not been shown to be effective in this pathology [3]. Due to the limited efficacy of chemotherapy in the context of metastatic disease, diverse therapeutic strategies have been explored. Initially, Interferon Alpha was studied as a potential treatment option for these patients, demonstrating a marginally improved response when compared to symptomatic treatment, albeit still characterised by a notably poor outcome, with a median survival of only 8.5 months [6]. In light of these considerations and taking into account the upregulated expression of VEGF in this disease, sunitinib, a first-generation TKI, was then studied and compared to Interferon Alpha therapy in patients with metastatic ccRCC, revealing a significantly superior response in terms of progression-free survival (PFS) and response rates (RR) [7]. Since then, new generations of TKI have been developed, including axinitinib, tivozanib and cabozantinib, all of which have exhibited favourable results in numerous phase III clinical trials [8–10]. In recent years, immunotherapy has emerged as a viable therapeutic option for the management of these patients. In fact, a phase III clinical trial demonstrated superior PFS and OS in patients with metastatic ccRCC when treated with the monoclonal antibody pembrolizumab in conjunction with the TKI lenvatinib in comparison with therapy with sunitinib alone [11].

## Case presentation

We present the case of a 61-year-old male patient with metastatic ccRCC treated at our institution, Fundación Santa Fe de Bogotá, after three prior lines of treatment at another institution. Notably, the patient had a background of chronic kidney failure. In his earlier years, he had a smoking history of 25 pack-years and had a history of alcohol consumption.

The patient was initially diagnosed with localised, stage 2 (T2aN0M0), right-sided ccRCC, in September 2009 outside our institution, as an incidental finding on an abdominal ultrasound as part of a routine medical examination. He underwent a successful right nephrectomy at another institution in Bogotá, with the histological analysis revealing polygonal cells arranged in cord-like patterns with enlarged and clear cytoplasm, with uninvolved margins, consistent with ccRCC. The patient subsequently continued to attend follow-up visits, with sustained remission until September 2015 when the patient presented with lumbar pain. An imaging study was performed, which suggested disease recurrence involving the L1 level of the lumbar spine and the psoas muscle. A psoas biopsy confirmed ccRCC relapse. The patient was treated with resection of the L1 vertebral body along with right lateral fixation between T12 and L2, followed by radiotherapy. From January to April 2016, he received sunitinib as maintenance therapy; this treatment was discontinued due to pulmonary thromboembolism and progression given by lung metastases in CT imaging. However, there was no evidence of toxicity. Low-molecular-weight heparin was initiated for anticoagulation purposes, while sunitinib was replaced with everolimus as second-line therapy, starting in April 2016.

**Figure 1.**
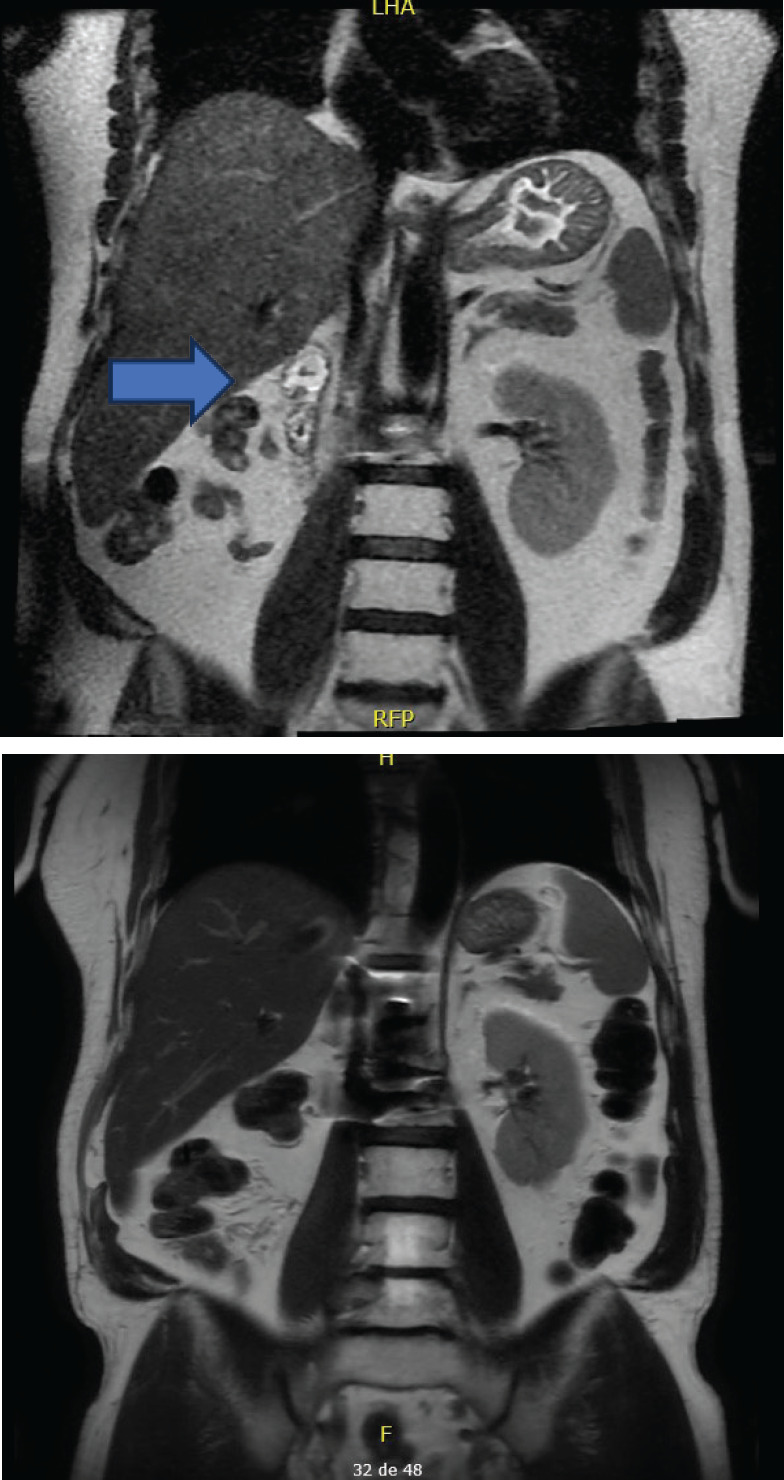
Evolution of the patient’s MRI over different time periods. Left: Abdominal MRI at the beginning of immunomodulatory therapy (30 December 2016) – showing post-right nephrectomy and two small solid nodules with neoplastic appearance found in the right renal fossa. Right: Abdominal MRI after 50 cycles of immunomodulatory therapy (8 July 2022) – showing a complete response with no secondary neoplastic involvement observed in the abdomen and pelvis.

**Figure 2.**
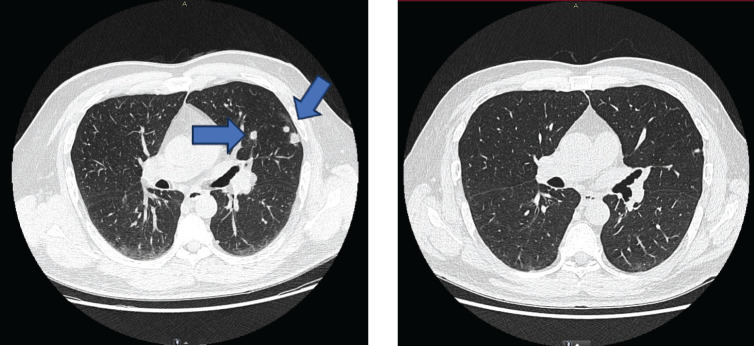
Evolution of the patient's computed tomography scans over different time periods. Left: CT scan at the beginning of immunomodulatory therapy (30 December 2016) – showing multiple solid pulmonary nodules (8–13 mm) predominantly in the peripheral left mid upper and lower lobes and left hilar adenopathies (15–17 mm). Right: CT scan (8 July 2022) – showing complete response of thoracic lesions after 50 cycles of immunomodulatory therapy.

There was no treatment-related toxicity. However, in November 2016, follow-up imaging, including bone scintigraphy and right ankle magnetic resonance imaging (MRI), were ordered due to right lower extremity oedema and pain. The gammagraphy results showed compromise of the remaining L1 spinous process and pathological L2 fracture, as well as secondary neoplastic bone infiltration of the hip, indicating new bone progression. Due to treatment failure, it was decided to discontinue everolimus. The patient received spinal, hip and ankle radiation therapy, this time with a palliative intent, until December 2016. In January 2017, the patient was referred to our service for further treatment. Extension studies were conducted, showing multiple bone metastases in the thoracic vertebrae, knee and right hip, as well as metastatic compromise of the lung, mediastinum, and retroperitoneal right renal fossa (Figures 1 and 2). Furthermore, the retroperitoneal mass biopsy confirmed the presence of ccRCC. The patient had an Eastern Cooperative Oncology Group performance status of 0 and a favourable International Metastatic Renal Cell Carcinoma Database Consortium (IMDC) index. Therefore, the decision was made to initiate a third-line pharmacologic treatment with nivolumab, beginning in March 2017. Concomitantly, given the vertebral and hip compromise, the patient received radiation therapy once again.

After the sixth cycle of immunotherapy, in July 2017, CT imaging evidenced resolution of the mediastinal adenopathies and reduction in both the size and number of the pulmonary nodules, while MRI imaging showed a decrease in the size of the bony compromise and stable abdominal lesions, exhibiting partial response for the first time since the onset of the metastatic disease. The patient consistently attended follow-up appointments, and there were no clinical or imaging changes in the disease until June 2019, after 35 cycles. At this time, on-going response to nivolumab treatment was evidenced by whole-body MRI, demonstrating a complete response of the previous intra-abdominal and pulmonary lesions along with a stable metastatic bone disease.

Later, upon cycle 44 of nivolumab, in December 2020, the patient was hospitalised for SARS-COV2 pneumonia. While in the hospital, he experienced an acute renal injury and required supportive care in the critical care unit, including non-invasive ventilation due to hypoxia. As a result, the administration of nivolumab was discontinued. After 20 days, he was discharged home. Simultaneously, follow-up studies were conducted, which showed stable disease. Immunotherapy was resumed 4 months later in April 2021, with appropriate tolerance.

During the following visits in the subsequent year, the patient continued with stable disease until July 2022, when complete remission was documented by the complete absence of hypermetabolic lesions on positron emission tomography-computed tomography (PET-CT). Notably, this was the first time in which imaging did not reveal any lesions since the initial documentation of metastatic disease (Figures 1 and 2).

**Figure 3.**
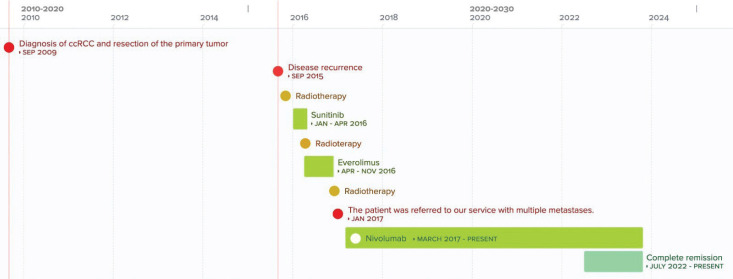
Chronological overview of key patient treatments and outcomes.

In April 2023, the patient completed 75 months of follow-up under our care. Follow-up studies including bone gammagraphy and lumbar spine MRI show no changes compared to previous studies. To date, the patient has received 66 cycles of immunotherapy without experiencing adverse effects and has sustained complete remission (Figure 3).

## Discussion

Over the past two decades, TKIs have played a central role in the clinical management of patients with metastatic ccRCC [12]. Nevertheless, recent advancements in our understanding of the tumour microenvironment and the immunogenic profile of ccRCC have laid the groundwork for novel therapeutic strategies [13]. One main mechanism used by malignant cells in this condition is T-cell function inhibition, achieved either through the expression of PD-L1 and PD-L2, which interact with the PD-1 receptor on T-cells, or via the expression of B7-H3, which binds to cytotoxic T-lymphocyte antigen 4 (CTLA-4), functioning as negative regulators of T-cell immune function. Therefore, the use of monoclonal antibodies that inhibit these receptors represents a viable strategy to restore the patient's tumour-specific T-cell response [14]. Since the advent of ICI in the management of patients with ccRCC, different pivotal studies have been conducted to measure their effectiveness [12].

In the context of systemic treatment for metastatic ccRCC, the European Society for Medical Oncology guidelines recommend, as first-line therapy, the use of PD-1 inhibitors, either in combination with TKI’s or CTLA-4 inhibitors, regardless of the risk groups established by the IMDC [15]. Specific recommended combinations include axitinib-pembrolizumab (KEYNOTE-426), cabozantinib-nivolumab (CheckMate 9ER), and, more recently, in accordance with the results of the CLEAR trial, lenvatinib-pembrolizumab. This last combination significantly demonstrated improvement in OS, RR, and PFS when compared to sunitinib (TKI) monotherapy [16–18]. While there is currently no preference for a particular combination among patients in the IMDC favourable risk group, there is evidence that monotherapy with sunitinib (TKI) was inferior to combination therapies including ICIs in terms of PFS, however, no differences in OS have been observed, likely attributed to the relatively limited duration of follow-up in the available data [15]. On the other hand, for patients in the intermediate and poor risk groups, the combination of ipilimumab (Anti-CLTA-4)-nivolumab (anti-PD-1) is recommended as first-line treatment [15]. Concerning second-line treatment, the recommendation is based on TKI's, although these data come from studies with heterogeneous populations and small sample sizes [15, 19–21].

With regards to the use of ICIs in patients who have experienced disease progression following two lines of systemic therapy, the evidence relies on the CheckMate 025 study. This randomised phase III clinical trial compared nivolumab versus everolimus in patients who had previously received systemic treatment for advanced ccRCC. Inclusion criteria specified a Karnofsky performance status of at least 70 upon study enrolment and disease progression during or after the last treatment regimen while excluding patients with central nervous system metastasis, previous treatment with an mTOR inhibitor, or a condition requiring treatment with glucocorticoids [22]. The study results favoured nivolumab in terms of OS (25 versus 19.6 months), response rate (25% versus 5%) and showed lower incidence of grade 3–4 treatment-related adverse events in the nivolumab group (19% versus 37%). No statistically significant difference was observed in PFS between the nivolumab and everolimus cohorts, with median PFS values of 4.6 and 4.4 months, respectively [22].

Subsequently, the NORA study evaluated monotherapy with nivolumab with the aim of capturing real-world data to complement the CheckMate 025 study, concluding that efficacy and safety were in line with the pivotal clinical trial and supporting the use of nivolumab after prior systemic therapy in a broad population of ccRCC patients [23]. Based on these results, monotherapy with nivolumab can be considered a reasonable approach for the management of advanced renal cell carcinoma following progression after prior systemic therapy.

We present the case of a 61-year-old male at diagnosis, who had received two previous systemic treatment regimens for metastatic ccRCC prior to initiating nivolumab monotherapy at our institution. This case exhibits several noteworthy characteristics. Notably, it offers insight into the broader applicability of the findings observed in the CHECKMATE 025 study. This is due to the fact that our patient is not within the study's defined population, given his prior treatment with an mTOR inhibitor before initiating nivolumab therapy, a condition that led to his exclusion from this study. Other remarkable findings of this case are the exceptional OS and PFS of the patient, which have been notably higher than the median values in the mentioned studies. Indeed, the Checkmate 025 study reported a median OS of 25 months and a median PFS of 4.6 months, while, in our patient's case, following the initiation of nivolumab therapy, the current OS stands at 75 months, with a corresponding PFS of 70 months [22]. However, it should be noted that none of the published studies report a follow-up of more than 5 years, so our case may represent an incentive for a longer follow-up period in these patients, in whom we believe OS with ICI monotherapy may be higher than what is reported in current literature.

The patient had previously undergone two systemic treatment regimens, both aligned with the guidelines recommended for his IMDC risk group. Specifically, the patient received sunitinib (TKI) as a first-line therapy, followed by everolimus (mTOR inhibitor) as the second-line treatment, before commencing nivolumab therapy. However, it is noteworthy that, following the documentation of metastasis, nivolumab monotherapy emerged as the sole treatment regimen exhibiting any measure of disease response, ultimately achieving, and maintaining a state of complete remission. This is of profound value, especially considering the extraordinary rarity of this event, observed in only 1% of patients subjected to nivolumab monotherapy in the Checkmate025 study [22].

On the other hand, an area of concern that should be addressed is the potential for toxicity, especially considering that the patient has been receiving ICI therapy for 6 years. Adverse events related to the immune system have been documented in 70% of the patients treated with anti-PD-1/PD-L1 agents [24]. The most frequently observed adverse events involve the skin, such as mild itching or rash, followed by gastrointestinal issues, often presenting as diarrhea and colitis. The third most common are endocrine, including thyroid dysfunction, pituitary inflammation, and adrenal insufficiency. Musculoskeletal and ocular toxicities, such as mild joint or muscle pain and mild dry eye syndrome or uveitis, are also commonly reported [24]. Nivolumab-specific adverse events were reported in the CHECKMATE025 study, with 79% of patients experiencing them. Fatigue was the most frequently reported (33%), followed by nausea (14%), itching (14%), diarrhoea (12%), loss of appetite (12%), and rash (10%) [22]. Our patient has exhibited good tolerance to nivolumab treatment, with no grade 3–4 adverse events occurring to date. The only significant occurrences took place following over 4 years after the start of nivolumab treatment, occurring after the 52nd and 54th treatment cycles, manifesting as a transient episode of fatigue and abdominal discomfort, respectively. In both instances, symptomatic management was implemented, resulting in subsequent improvement as confirmed by follow-up assessments. Throughout the ensuing cycles, no substantial new developments emerged, and the patient consistently maintained excellent medication tolerance.

In cases such as the one presented in this article; the term 'long survivor' has been used to describe a group of patients undergoing immunotherapy who have exceeded expectations. This leads to questions regarding whether treatment should be discontinued after a patient achieves sustained stable illness or remission, or if therapy should be continued indefinitely. According to the guidelines, discontinuing ICIs should be considered after 2 years of therapy; however, this recommendation lacks specificity [15]. Due to the relatively recent use of ICI for the management of ccRCC, there are very few cases in the literature of suspension of immunotherapy with persistence of the response [25]. Several case reports have been documented on patients with renal carcinoma experiencing ‘long survivor’ with other types of systemic treatments, including TKI (pazopanib, axitinib) [26–28], mTOR inhibitors (everolimus), and ICI’s (pembrolizumab) [28], cases related to ‘long survival’ with nivolumab are scarce. In the literature, there is a case report of a patient diagnosed with ccRCC who initially underwent treatment with sunitinib. However, due to intracardiac metastasis, the treatment was switched to nivolumab, resulting in a reduction in the size of the metastasis that remained stable for 12 months while continuing the nivolumab treatment [29]. Another case report described a patient who received treatment with a combination of nivolumab and ipilimumab, resulting in a partial response for 15 months [30]. However, after this period, the disease progressed, and the patient passed away [30]. The case presented adds to this limited evidence and represents, to the best of our knowledge, the longest documented OS in current literature for patients treated with nivolumab monotherapy for metastatic ccRCC that had previously progressed after two lines of systemic therapy.

Moreover, another factor to consider when contemplating extended nivolumab treatment is the substantial expense associated with this therapy. Nivolumab is a high-cost medication, with price per treatment cycle ranging from $282.97 to $353.71. This cost may not be readily affordable, especially when compared to average incomes and the current minimum wage in our country, which stands at $273.54 per month. However, in Colombia, more than 95% of the population is affiliated to a health promoting entity (HPE), where the cost of medical care and treatment is adjusted based on their monthly incomes, making it more affordable [31]. In the case of our patient, he was affiliated to a HPE, which covered a substantial portion of the treatment costs.

In light of all these factors, there is still a major therapeutic challenge for the treating physician with regard to when it is appropriate to suspend therapy [25]. Finally, this is a case that supports the use of ICI as monotherapy for patients with metastatic ccRCC, a highly effective, relatively safe option, that has dramatically improved patient survival and allowed us to start thinking about ‘long survivors’, even in patients with multiple metastatic involvement and who failed to respond to previous systemic therapies, as was the case presented here.

## Conclusion

Metastatic ccRCC is associated with a very poor prognosis. Historically, many different therapeutic agents have been proposed for the management of these patients, with the most recent advances pointing towards an important role of ICI. This case report highlights the efficacy and safety of ICI monotherapy in the management of metastatic ccRCC. It suggests a potential for achievement of sustained complete remission in patients who did not respond to prior systemic therapies. The prolonged OS and PFS of the patient further support the potential benefits of ICI monotherapy in extending the life expectancy of patients with advanced ccRCC. Given the limited literature available on the long-term use of ICI monotherapy in patients with ccRCC, this case contributes to the understanding of long-term survival and management strategies in these patients. The case raises important questions about the discontinuation of therapy after achieving sustained remission, warranting further research in this area.

## Conflicts of interest

The authors declare that they have no conflicts of interest.

## Funding

The authors have not received any kind of funding for the research and writing of this paper.

## Consent for publication

Consent for publication was obtained from the patient.
